# Enhancing Radiotherapy for Locally Advanced Non-Small Cell Lung Cancer Patients with iCE, a Novel System for Automated Multi-Criterial Treatment Planning Including Beam Angle Optimization

**DOI:** 10.3390/cancers13225683

**Published:** 2021-11-13

**Authors:** Kristine Fjellanger, Liv Bolstad Hysing, Ben J. M. Heijmen, Helge Egil Seime Pettersen, Inger Marie Sandvik, Turid Husevåg Sulen, Sebastiaan Breedveld, Linda Rossi

**Affiliations:** 1 Department of Oncology and Medical Physics, Haukeland University Hospital, 5021 Bergen, Norway; liv.bolstad.hysing@helse-bergen.no (L.B.H.); helge.egil.seime.pettersen@helse-bergen.no (H.E.S.P.); inger.marie.sandvik@helse-bergen.no (I.M.S.); turid.husevag.sulen@helse-bergen.no (T.H.S.); 2 Institute of Physics and Technology, University of Bergen, 5007 Bergen, Norway; 3 Department of Radiotherapy, Erasmus University Medical Center (Erasmus MC), 3015 GD Rotterdam, The Netherlands; b.heijmen@erasmusmc.nl (B.J.M.H.); s.breedveld@erasmusmc.nl (S.B.); l.rossi@erasmusmc.nl (L.R.)

**Keywords:** autoplanning, automated treatment planning, beam angle optimization (BAO), beam configuration, Erasmus-iCycle and Eclipse, iCE, locally advanced non-small cell lung cancer (LA-NSCLC), intensity-modulated radiotherapy (IMRT), radiotherapy

## Abstract

**Simple Summary:**

In treatment planning for intensity-modulated radiotherapy (IMRT), optimization objectives and beam angle settings are individualized for the anatomy of each patient. This is a complex interactive process that is usually performed by a treatment planner. In this study, a novel system for automated optimization of IMRT plans with integrated beam angle optimization (BAO) was developed, and used to systematically investigate the impact of selected beam angles on treatment plan quality for locally advanced non-small cell lung cancer (LA-NSCLC). Automatically generated plans were of a higher quality than the manually generated, clinically delivered plans, while dramatically reducing the planning workload. The study demonstrates the potential for automated planning with integrated BAO to enhance radiotherapy for LA-NSCLC patients.

**Abstract:**

In this study, the novel iCE radiotherapy treatment planning system (TPS) for automated multi-criterial planning with integrated beam angle optimization (BAO) was developed, and applied to optimize organ at risk (OAR) sparing and systematically investigate the impact of beam angles on radiotherapy dose in locally advanced non-small cell lung cancer (LA-NSCLC). iCE consists of an in-house, sophisticated multi-criterial optimizer with integrated BAO, coupled to a broadly used commercial TPS. The in-house optimizer performs fluence map optimization to automatically generate an intensity-modulated radiotherapy (IMRT) plan with optimal beam angles for each patient. The obtained angles and dose-volume histograms are then used to automatically generate the final deliverable plan with the commercial TPS. For the majority of 26 LA-NSCLC patients, iCE achieved improved heart and esophagus sparing compared to the manually created clinical plans, with significant reductions in the median heart D_mean_ (8.1 vs. 9.0 Gy, *p* = 0.02) and esophagus D_mean_ (18.5 vs. 20.3 Gy, *p* = 0.02), and reductions of up to 6.7 Gy and 5.8 Gy for individual patients. iCE was superior to automated planning using manually selected beam angles. Differences in the OAR doses of iCE plans with 6 beams compared to 4 and 8 beams were statistically significant overall, but highly patient-specific. In conclusion, automated planning with integrated BAO can further enhance and individualize radiotherapy for LA-NSCLC.

## 1. Introduction

The standard treatment for locally advanced non-small cell lung cancer (LA-NSCLC) is concurrent chemoradiotherapy [1]. Intensity-modulated radiotherapy (IMRT) is the state-of-the-art radiation technique, allowing optimal shaping of the delivered dose to the target. IMRT has improved outcomes compared to conformal radiotherapy, but side effects are still common and potentially severe [2]. Decreasing the radiation dose to organs at risk (OARs) is desirable both for toxicity reduction and the potential for dose escalation [1,3].

The aim of radiotherapy treatment planning is to establish treatment unit settings for each patient that will result in a high-quality dose distribution, i.e., a high dose inside the target volume and limited dose outside, especially in the OARs. IMRT treatment plans are generated using a cost function that defines the planning objectives for the target, OARs and other tissues, and their relative weights. A mathematical optimizer derives patient-specific beam intensity profiles that minimize the cost function value. In manual IMRT treatment planning, the general approach is to begin the plan generation for a new patient using a tumor-site-specific template for the cost function. In an iterative trial-and-error process, the objectives and weights are then adapted by the manual planner to account for the patient’s anatomy. For fixed-beam IMRT treatment in the thorax, the beam angles should also be individually tuned, as IMRT with carefully selected beam angles can reduce OAR doses compared to volumetric modulated arc therapy (VMAT) or IMRT with non-optimized beam angles [4]. In clinical routine, time pressure limits the possibility to test many different objective and beam angle settings, and the planner’s experience or skills may affect the quality of the plans. A number of studies have demonstrated that manually created plans may be suboptimal [5,6,7,8,9].

In recent years, several systems for automated treatment planning have been presented. Compared to manual planning, automated planning can considerably increase the plan quality while dramatically reducing the planning workload [7].

Erasmus-iCycle, developed at the Erasmus University Medical Center (Erasmus MC, Rotterdam, The Netherlands), is a system for automated multi-criterial treatment planning that can generate beam profiles for pre-selected beam directions, but it also features integrated beam profile and beam angle optimization (BAO) [10,11,12,13,14,15,16]. With appropriate treatment-site-specific configuration (creation of a “wish-list”), the generated Pareto-optimal plans are also clinically favorable. Erasmus-iCycle generates plans using fluence map optimization (FMO), but there is no consecutive segmentation, and therefore the plans are not directly deliverable. For the generation of clinically deliverable plans at Erasmus MC, Erasmus-iCycle is used as a pre-optimizer, with consecutive automatic reconstruction of FMO plans into segmented plans in the Monaco treatment planning system (TPS) (Elekta AB, Stockholm, Sweden). For this purpose, patient-specific Monaco optimization templates are created based on the achieved constraint and objective values in the Erasmus-iCycle FMO plan. Several publications have demonstrated the effectiveness of this approach in reducing OAR doses and planning time for different treatment sites [17,18,19,20,21], including VMAT of LA-NSCLC [8]. The Erasmus-iCycle wish-list-based lexicographic optimization approach for automated multi-criterial treatment planning has recently been adopted by a commercial party [22].

The Eclipse TPS (Varian Medical Systems, Inc., Palo Alto, CA, USA) features the RapidPlan system for automated knowledge-based treatment planning, whereby a library of previous treatment plans is used to predict feasible patient-specific OAR dose-volume histograms (DVHs) for new patients [23]. The predicted DVHs are converted into optimization parameters for automatic generation of deliverable treatment plans. A limitation of RapidPlan for automated IMRT planning for LA-NSCLC is that it does not feature patient-specific BAO. Therefore, the beam configuration must be determined manually by trial-and-error.

While treatment planning systems featuring advanced BAO are not commercially available, BAO methods have been investigated for their use in radiotherapy of lung cancer. Yuan et al. used a wide range of lung tumors to tune a BAO system for coplanar configurations, and applied it for more complex, non-coplanar plans [24]. Amit et al. presented a learning-based method which was applied for various thoracic indications [25]. In both studies, beam angle and beam profile optimization were not integrated; first, patient-specific beam angles were established, followed by IMRT optimization for the selected (fixed) angles. The results showed that plans with optimized beam angles had similar quality to clinical plans with manually selected beam angles.

The aim in this study was to automatically create IMRT plans with integrated optimization of beam angles for a prospective database of LA-NSCLC patients, in order to improve plan quality compared to manually created plans and to investigate the impact of beam angles on plan quality. For this purpose, Erasmus-**iC**ycle was coupled with **E**clipse to establish the novel “**iCE**” system. In iCE, Erasmus-iCycle is used as a pre-optimizer to automatically generate an initial Pareto-optimal FMO treatment plan with optimized beam angles for each patient. The OAR DVHs of this plan are converted into patient-specific optimization parameters for automated generation of the final deliverable plan in Eclipse. Both iCE plans and autoplans using the manually selected beam angles were compared to the manually generated, clinically delivered plans. Finally, iCE was used to investigate the effect of changing the number of IMRT beams.

## 2. Materials and Methods

### 2.1. Patients and Clinical Treatment Planning

Twenty-five consecutive patients with stage IIB-IIIC non-small cell lung cancer were prospectively included in this study. One patient with stage IVA, who had a single brain metastasis surgically removed prior to radiotherapy, received radiotherapy according to the protocol for LA-NSCLC and was also included. All patients received IMRT and concurrent or sequential chemotherapy at Haukeland University Hospital (HUH) between October 2019 and August 2021. The study was approved by the regional committee for medical and health research ethics (protocol code 2019/749) and all participants provided their informed consent.

All patients had both a 10-phase 4DCT and a deep inspiration breath-hold (DIBH) CT at planning. Treatment plans were created on the average intensity projection (AIP) of the 4DCT and treatment was given in free breathing (FB) conditions as a standard. However, for patients with very large breathing motion or where lung dose constraints could not be met with the AIP, the treatment plans were created on the DIBH CT instead (four patients).

The responsible oncologist delineated the gross tumor volumes (GTVs) for the primary tumor and lymph nodes according to ESTRO guidelines [26]. For FB treatment, the internal GTVs (IGTVs) included the GTV positions on all 4DCT phases. For DIBH treatment, three repeated DIBH scans were taken at planning and the IGTVs encompassed the GTV positions on all three scans. The clinical target volume (CTV) was defined by expanding the IGTV by 5 mm without extending into uninvolved organs such as bone, heart, esophagus and major vessels. A 5 mm isotropic margin from the CTV was used to define the planning target volume (PTV). As OARs, the lungs, heart, esophagus, spinal canal and brachial plexus (if relevant) were delineated according to RTOG guidelines [27].

The clinical plans (CLIN) were manually created by expert planners in Eclipse v. 15.6 or 16.1 using the Photon Optimizer algorithm for optimization and the Acuros External Beam algorithm for dose calculation. All plans included 6 coplanar IMRT beams with beam angles based on a template that was individually adapted. In accordance with national guidelines, the prescribed dose was 60 or 66 Gy for concomitant treatment (depending on lung function, lung dose and proximity of the brachial plexus to the PTV) and 70 Gy for sequential treatment, all administered in 2 Gy fractions. The plans were normalized to the median dose in the PTV. The dose constraints applied for planning are provided in Table 1.

### 2.2. iCE Treatment Planning

#### 2.2.1. Erasmus-iCycle Wish-List Creation and FMO Plan Generation

A detailed description of Erasmus-iCycle functionality and wish-list creation can be found elsewhere [10,28]. In this study, an Erasmus-iCycle wish-list for LA-NSCLC was established and tuned according to clinical priorities at HUH. An oncologist (IMS) and an expert planner (THS) were involved in the evaluation of treatment plans during this process. In the first phase of tuning, five of the patients treated in FB with 66 Gy were used. The PTV coverage and high dose conformity were kept similar to the clinical plans, while the dose to the OARs and undefined normal tissue was minimized. The wish-list was then applied for an additional four patients with a 60 Gy prescription and/or DIBH CT, and some final adjustments were made.

In the final wish-list, hard constraints were used for the maximum dose to the spinal canal, brachial plexus and PTV, and for an external ring to prevent high entrance doses. Target and OAR objectives were added with the following order of priority (in line with clinical priorities): PTV coverage, lung D_mean_, heart D_mean_ and esophagus D_mean_. In addition, constraints and objectives were applied for normal tissue at specified distances from the PTV to steer conformity. The full wish-list is provided in Table 2.

In the multi-criterial plan generation, the objective functions are consecutively minimized following the allotted priorities. After the minimization of an objective function, a constraint is added to the problem to avoid quality loss for this objective when minimizing the following lower priority objectives. Two rounds of consecutive minimizations of objective functions are applied. In the first round, the constraint that is added after an objective minimization is the Goal value (second to last column in Table 2), if it could be obtained, regardless of the possibility for further improvement. If the Goal value is not achieved, the obtained objective value, with some relaxation, is added as a constraint. In the second round, all objectives without a Sufficient value (last column in Table 2) are minimized to the fullest extent, following the order of priority. In this way, the Goal and Sufficient values make sure undesired and unnecessary greediness in minimizing objectives is avoided, leaving room for minimizing lower priority objectives [10].

The candidate beam angles for BAO were in the range of 140–40° for right-sided tumors and 320–220° for left-sided tumors with 5° spacing.

#### 2.2.2. Generation of the Final iCE Plan Based on an Initial Erasmus-iCycle FMO Plan

The Erasmus-iCycle FMO plan was used to create a patient-specific objective template for automated generation of the final deliverable plan in Eclipse. This was performed automatically by a script that moved information from the OAR DVHs in .csv format into an objective template in .xml format (see Supplementary Materials). For each OAR involved, a line objective was created. Line objectives are defined by a collection of dose-volume pairs and limit the dose for all volume levels [29]. The distance between dose-volume points defining the line was set to 0.7 Gy. For the PTV objectives, the same fixed settings as in the clinical plans were used. Priorities and normal tissue objective settings were kept constant after tuning based on the first five patients. Table 3 shows the final template that was used for all of the patients.

The generated objective templates as well as the optimized beam angles from Erasmus-iCycle were used for automated IMRT plan optimization in Eclipse, with no manual fine-tuning. The applied Eclipse version was the same as the version used for clinical planning. The final iCE plans were visually inspected to ensure that the high-dose conformity and the dose to undefined normal tissue was acceptable and comparable to the CLIN plans.

### 2.3. Comparison of iCE and CLIN Plans

For each patient, a 6-beam iCE plan (same beam number as clinically used) was generated, and compared to the corresponding CLIN plan using relevant dose-volume metrics for the target and OARs. The mean dose to the lungs, heart and esophagus are commonly reported parameters related to toxicity, and were therefore used for evaluation of per-patient differences. To separately assess the benefit of BAO in iCE, a second iCE plan (iCE_noBAO) was generated for each patient, using the same beam angles as the CLIN plan, i.e., BAO in iCE was switched off.

### 2.4. Planning Times

The manual planning time was estimated from discussions with three treatment planners involved in clinical LA-NSCLC planning. The hands-on time in iCE planning, related to starting autoplanning and transfer of data between systems, was recorded.

### 2.5. Dosimetric QA of Plan Deliverability

For 10 randomly selected patients, the deliverability of the iCE plans was evaluated following the current clinical quality assurance (QA) procedure for this diagnosis at HUH. Electronic Portal Imaging Device measurements were performed, followed by analysis in the Portal Dosimetry system in Eclipse. A gamma passing rate of 95%, with a global criterion of 3%/3 mm, was required. In addition, the number of monitor units (MUs) in CLIN and iCE plans were compared.

### 2.6. 6-Beam vs. 4- and 8-Beam iCE

In addition to the 6-beam plans, iCE was also used to generate plans with 4 and 8 beams, to validate the clinical use of 6 beams for all patients. Relevant target and OAR dose-volume metrics were compared for the different numbers of beams.

### 2.7. Statistical Analysis

The two-tailed Wilcoxon signed-rank test was used for the statistical testing of dose-volume parameters for CLIN vs. iCE and iCE_noBAO plans, and 6-beam vs. 4- and 8-beam plans. *p*-values ≤ 0.05 were considered as statistically significant.

## 3. Results

### 3.1. Patients

Among the 26 included patients, 21 had both a primary tumor and lymph nodes in the target volume, one had only lymph nodes and four had only a primary tumor. The distribution of stages, primary tumor locations, dose prescriptions and timing of the chemotherapy are summarized in Table 4. The average PTV volume was 395 cm^3^ (range 138–1715 cm^3^).

### 3.2. Comparison of iCE and CLIN Plans

**Figure 1:**
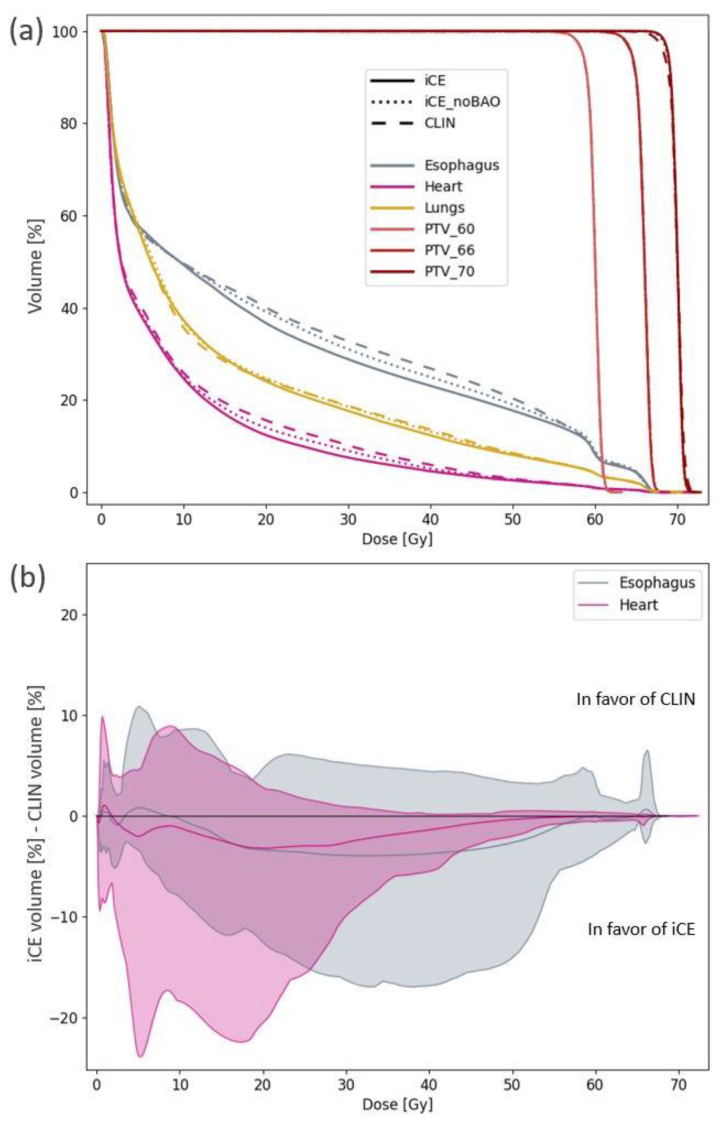
(**a**) Population average DVHs for PTV and OARs for CLIN, iCE and iCE_noBAO plans. 10 patients had PTV_60, 15 had PTV_66 and 1 had PTV_70. (**b**) Average DVH differences between CLIN and iCE plans for heart and esophagus (central bold lines) with 95% confidence intervals (shaded areas). For lungs, the differences were small and clinically insignificant.

Overall, the iCE plans were clearly superior to the CLIN plans (Table 5, Figure 1). While the target coverage and lung dose were similar, the median heart D_mean_ was reduced from 9.0 Gy to 8.1 Gy (*p* = 0.02), the median esophagus D_mean_ from 20.3 Gy to 18.5 Gy (*p* = 0.02), the median heart V_30Gy_ from 11.0% to 6.2% (*p* = 0.002) and the median esophagus V_20Gy_ from 38.4% to 36.8% (*p* = 0.008) for iCE compared with CLIN. The maximum dose to the brachial plexus and patient body followed the clinic’s requirements for all plans. The maximum dose to the spinal canal slightly exceeded the limit for two patients in both the CLIN and iCE plans, but these violations were located in very small volumes at the edge of the contour and were therefore found to be clinically acceptable.

**Figure 2:**
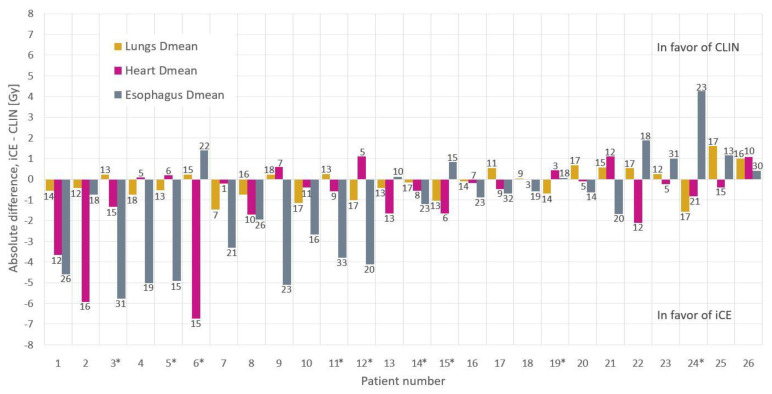
Differences in OAR mean doses between CLIN and iCE plans per patient. The numbers on the bars indicate the D_mean_ values [Gy] in the CLIN plans. The patients are sorted according to the sum of differences for all OARs. Patients marked with * were used in wish-list tuning.

The iCE plans spared the heart and esophagus for most dose levels as compared to CLIN (Figure 1b). The 95% confidence interval shows the large advantage of using iCE for some patients, with reductions of more than 15 percentage points in the heart volume receiving 5–25 Gy, and the esophagus volume receiving 25–50 Gy. The potential for substantial OAR sparing with iCE for individual patients is also evident in Figure 2, which shows reductions of more than 5 Gy in the D_mean_ of both the heart and esophagus compared to CLIN. iCE reduced the heart D_mean_ for 19/26 patients and the esophagus D_mean_ for 17/26 patients. The differences in the mean lung dose were small (Figure 1a and Figure 2).

**Figure 3:**
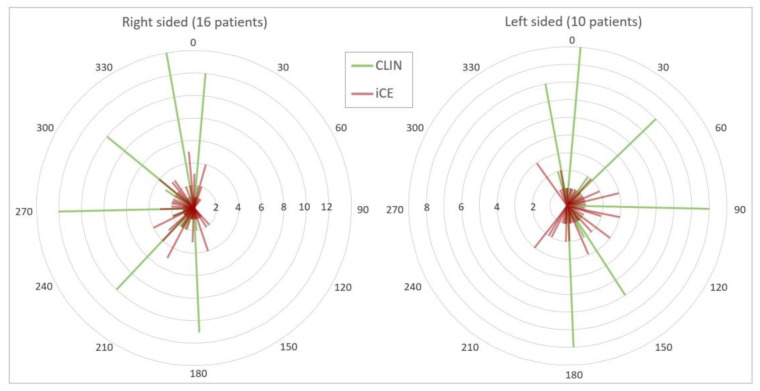
Selected beam angles in the CLIN plans (green) and optimized beam angles in the iCE plans (red) for patients with right-sided and left-sided tumors. Angles are rounded to the nearest 5 degrees. The number of patients is shown on the radial axis, and the angular axis shows beam angle in degrees.

**Figure 4:**
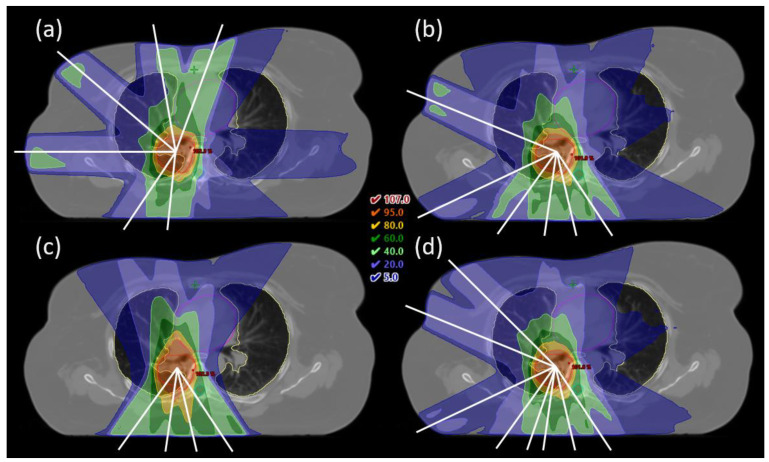
(**a**) CLIN plan with manually selected beam angles for patient 1. (**b**–**d**) iCE plans with 6, 4 and 8 optimized beam angles for the same patient. Dose is shown relative to the prescribed dose (66 Gy). Contours are the PTV (red), lungs (yellow), heart (magenta), esophagus (grey) and spinal cord (cyan), and beam angles are indicated by white lines.

**Figure A1:**
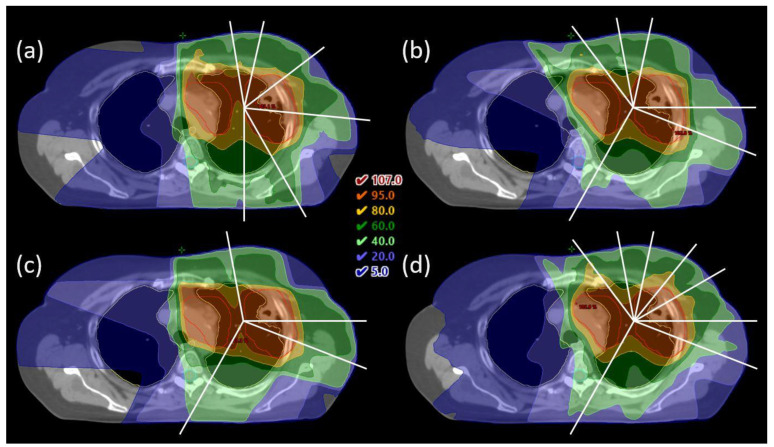
(**a**) CLIN plan with manually selected beam angles for patient 4, with a frontal tumor and lymph nodes. (**b**–**d**) iCE plans with 6, 4 and 8 optimized beam angles for the same patient. Dose is shown relative to the prescribed dose (66 Gy). Contours are the PTV (red), lungs (yellow), esophagus (grey) and spinal cord (cyan), and beam angles are indicated by white lines. For this patient, the optimized beam angles in the 6- and 8-beam plans enabled sparing of the esophagus.

**Figure A2:**
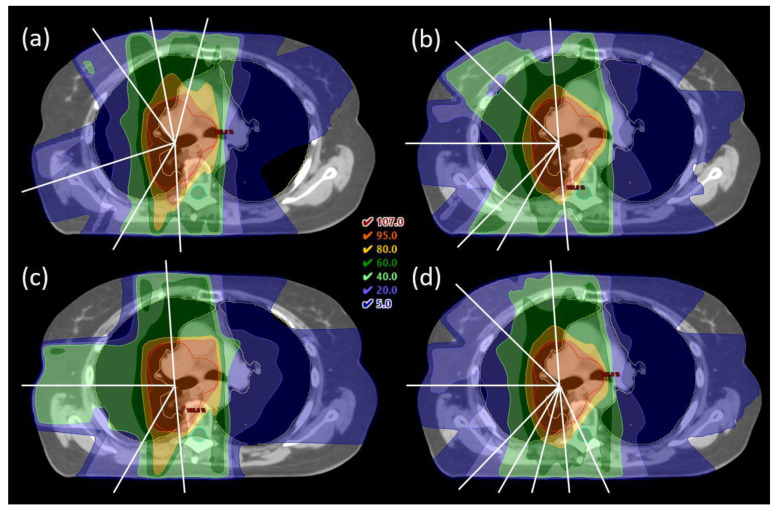
(**a**) CLIN plan with manually selected beam angles for patient 25, with a centrally located tumor. (**b**–**d**) iCE plans with 6, 4 and 8 optimized beam angles for the same patient. Dose is shown relative to the prescribed dose (66 Gy). Contours are the PTV (red), lungs (yellow), esophagus (grey) and spinal cord (cyan), and beam angles are indicated by white lines. For this patient, the distribution of high dose in the CLIN plan was not as conformal as desired. The 6-beam iCE plan has slightly higher lung and esophagus D_mean_ and better high dose conformity.

In general, the beam configurations used in the CLIN plans had most weight on the anterior-posterior direction, with little variation in the angles chosen for each patient. In contrast, the optimized beam angles in the iCE plans were well dispersed across the candidate beam space, revealing a large difference in optimal angles between patients (Figure 3). In Figure 4a,b, the optimized beam configuration in the iCE plan for patient 1 is compared with the configuration in the CLIN plan. The clear differences between the setups result in considerable sparing of the heart and esophagus with iCE (see also Figure 2). Additional examples with different tumor locations are shown in the Appendix A (Figure A1 and Figure A2).

When switching off BAO in iCE (iCE_noBAO), the reductions compared to CLIN in heart V_30Gy_ and esophagus D_mean_ were smaller than with BAO, but remained statistically significant. Figure 1 and Table 5 illustrate that some OAR sparing is achieved with iCE_noBAO compared to CLIN, and adding BAO in iCE results in a further improvement.

### 3.3. Planning Times

The manual planning time for the CLIN plans was usually between 2 and 4 h, although it could vary from less than an hour to a full day depending on the complexity of the case. This mainly comprised hands-on time, including adjustments of the beam configuration and optimization objectives during repeated rounds of optimization. For iCE, hands-on time was less than 10 min. The Erasmus-iCycle calculation time for automated generation of 6-beam plans was 1.5–3 h (7–25 min without BAO), and automated generation of the final deliverable plan with Eclipse took around 5 min.

### 3.4. Dosimetric QA of Plan Deliverability

All fields in all measured plans (in total 60 fields) passed the clinical requirement in the Portal Dosimetry analysis, with an average passing rate of 99.97% (range 99.0–100%). The average number of MUs in the iCE plans was 778 (range 474–1323), compared to 687 (range 406–1159) in the CLIN plans.

### 3.5. 6-Beam vs. 4- and 8-Beam iCE

**Figure 5:**
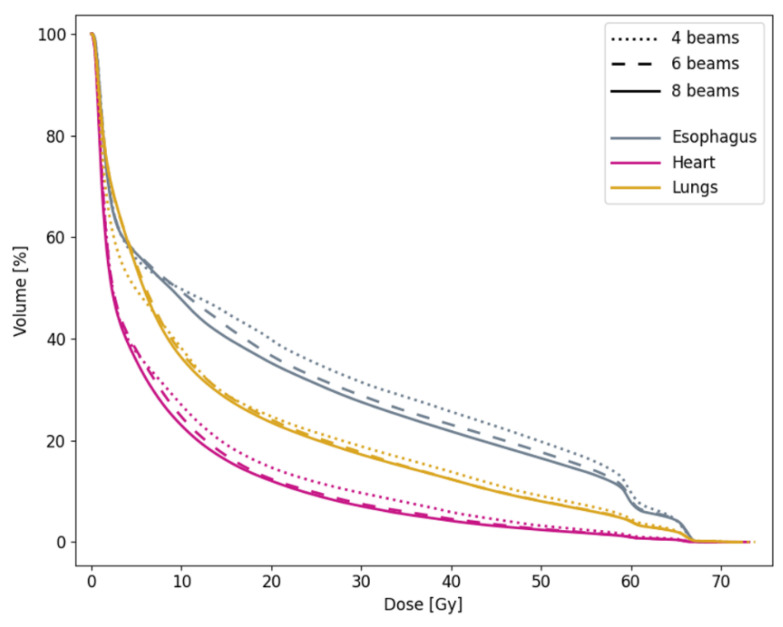
Population average DVHs for OARs in 4-, 6- and 8- beam iCE plans with optimized beam angles.

While 6- and 8-beam iCE plans generally fulfilled the clinical dose constraints, not all 4-beam plans would have been acceptable due to a D_max_ in the spinal canal or patient body above the requirement (Table A1). High- and medium-dose conformity was also generally worse with 4 beams than with 6 or 8, as visible in Figure 4, Figure A1 and Figure A2. The target coverage was similar regardless of the number of beams. Reducing the number of beams from 6 to 4 led to a median increase in D_mean_ of 0.7 Gy to the heart (*p* = 0.007) and 1.2 Gy to the esophagus (*p* = 0.02). Increasing the number of beams from 6 to 8 had a smaller, but still significant, benefit of 0.3 Gy to the heart (*p* = 0.02) and 0.7 Gy to the esophagus (*p* < 0.001) (Table 6 and Figure 5). The median D_mean_ for the lungs was similar for the different numbers of beams, but the DVH shows that 4-beam plans, on average, gave less low dose and more medium and high dose to the lungs compared with the 6- and 8-beam plans.

While the OAR doses overall decreased with an increasing number of beams, there was significant inter-patient variation (Table 6 and Table A1). With 8 beams, for 15/26 patients the D_mean_ was reduced for all of the evaluated OARs compared to 6 beams, while for the remainder of patients, the dose was increased for one of the OARs. Around half the patients had a reduced lung D_mean_ with 4 beams compared to 6 beams, but this came at the cost of a higher dose to the heart and/or esophagus. For patient 1, less spreading of the low dose with 4 beams resulted in a sparing in the lung D_mean_ of 2.5 Gy compared to 6 beams (Figure 4b–d). However, this was achieved at the cost of increased heart and esophagus D_mean_ of 1.8 Gy and 3.3 Gy respectively, and worse high-dose conformity. In the 8-beam plan, the lung D_mean_ was slightly higher than with 6 beams, while sparing of the heart and esophagus was improved. For some of the patients, changing the number of beams had little impact on the OAR doses; three patients had a change in the D_mean_ of less than 1 Gy for all OARs, both when reducing and when increasing the number of beams.

## 4. Discussion

The novel iCE system for automated multi-criterial treatment planning with integrated BAO was developed, and used to investigate opportunities for OAR sparing in LA-NSCLC patients, and to systematically investigate the impact of beam angles on the radiotherapy dose. Compared to clinical plans, significant reductions in heart D_mean_ and V_30Gy_ and esophagus D_mean_ and V_20Gy_ were achieved with iCE. When using iCE with integrated patient-specific BAO, OAR sparing was found to be superior compared to using iCE for clinically applied beam angles. Increasing the number of optimized beams can improve OAR sparing. On average, differences in OAR sparing were found to be larger between 4 and 6 beams than between 6 and 8. The impact of the beam number varied largely between patients, and for some patients, changing the number of beams did not have a clinically relevant impact on the dose to any OAR.

While the reductions with iCE compared to CLIN in median D_mean_ for OARs were 0.5–1.8 Gy, a substantial sparing of >5 Gy was seen for the heart and esophagus for individual patients. This confirms one of the main strengths of automated planning; the ability to achieve a more homogeneous plan quality by avoiding occasional highly suboptimal plans. Bradley et al. reported an association between increased heart V_30Gy_ and increased risk of death, and Dess et al. found that mean heart dose, V_5Gy_ and V_30Gy_ were associated with grade ≥3 cardiac events [31,32]. Wijsman et al. reported on a correlation between mean esophagus dose and grade ≥2 acute esophageal toxicity [33]. These correlations suggest that the observed reductions in heart D_mean_ and V_30Gy_ and esophagus D_mean_ with iCE compared to CLIN are of clinical importance. Only minor differences were observed for lung dose, possibly because the lungs received the most attention in clinical planning. The dose to the spinal canal and brachial plexus were not studied in detail, as reductions below the maximum dose constraints are not considered a priority. An additional advantage of iCE compared to manual planning is the dramatic decrease in hands-on planning time, as the trial-and-error process of finding objectives and beam angles manually is avoided.

A previous study using Erasmus-iCycle for LA-NSCLC found reductions in the average D_mean_ of the lungs, heart and esophagus of 0.9, 1.5 and 3.6 Gy for automatically generated VMAT plans compared to manually created IMRT plans [8]. Although not directly comparable to our results, as the dose prescription, clinical priorities, delivery technique and TPS differed, the potential for reducing OAR doses for most patients with automated planning was found in both studies. The same has been found for other autoplanning strategies, including knowledge-based planning in Eclipse and multi-criteria optimization in RayStation (RaySearch Laboratories, Stockholm, Sweden) [9,23]. These findings highlight the potential of improving the plan quality in LA-NSCLC by increasing the availability, integration and utilization of autoplanning systems.

None of the published studies on automated planning for LA-NSCLC included integrated BAO to systematically investigate the impact of beam angles on plan quality, as has been performed in this study. Fixed-beam IMRT has the potential to reduce low dose to the lungs compared to VMAT, but exploiting this requires individual selection of appropriate beam angles [4]. LA-NSCLC is a heterogeneous patient group, and the position of the tumor in relation to OARs strongly affects which beam angles are most beneficial. The beam configurations in the CLIN plans, based on a template with emphasis on the anterior-posterior direction and a couple of additional lateral fields, were well suited for central tumors. For frontal and dorsal tumors, more oblique configurations, sometimes with opposing fields to cover nodal volumes, were selected by iCE and could spare dose to the heart, esophagus and/or contralateral lung for specific patients. Clearly, beam angles should be optimized individually in IMRT for LA-NSCLC.

In general, 6-beam plans appear to be a reasonable choice for this patient group. In some situations, reducing the number of beams in a treatment plan might be desirable, e.g., when the treatment time is of particular concern due to large target volume, split fields, DIBH treatment or poor condition of the patient. Similarly, increasing the number of beams might be appropriate in some situations, e.g., if OAR doses are above constraints. With the iCE method, creating several treatment plans with different numbers of optimized beams can be performed automatically, allowing for individual assessment of alternative plans. Plans with different numbers of beams can be created in a single round of optimization in Erasmus-iCycle, only the reconstruction in Eclipse must be done separately. Note that not all of the 4-beam iCE plans would be clinically acceptable. The wish-list constraints for the D_max_ in the spinal canal and patient body as well as the high dose conformity were always achieved by default in Erasmus-iCycle. However, Eclipse struggled to reproduce the 4-beam dose distribution for some patients, and as hard constraints are not applied in Eclipse optimization, unacceptable plans could occur.

Increasing the number of beams on average improved OAR sparing, but the effect varied between patients. This is not surprising given the large heterogeneity in the anatomy of the LA-NSCLC patients. While the reasons behind the patient-specific differences were not investigated in detail, they appeared to be related to the OAR doses. Patients with high OAR doses had a greater benefit of increasing the number of beams than patients with low OAR doses. No impact of the size or location of the tumor was observed.

The average number of MUs was higher in the iCE plans than the CLIN plans (778 vs. 687). This could possibly be related to choices in the collimator angles, which were fixed at 2° in iCE plans, while in the CLIN plans, the collimator angle for each field was tuned to narrow the field size in the X direction, leading to larger field openings and fewer MUs. In our clinic, the enhanced MUs with iCE were not a concern, given the excellent deliverability of the plans. No restriction on the number of MUs was applied in the optimization of CLIN or iCE plans. Nonetheless, if desired, adjusting the collimator with regard to the optimized beam angles is a simple operation.

Different systems for automated treatment planning have different characteristics, advantages and limitations [7]. Advantages of Erasmus-iCycle are that configuration with a high-quality wish-list will result in optimal dose distributions according to the clinic’s priorities, and that few (or even zero) example plans are needed for the wish-list configuration. In many studies, Erasmus-iCycle plans proved superior to the example plans used for configuration [13,17,19,34,35]. A limitation is that the generated dose distributions are not directly deliverable. Therefore, translations into segmented treatment plans must be performed. In this study, the translations were performed by the Eclipse TPS, with use of line objectives that offer a simple way of controlling the entire dose-volume range simultaneously. It also makes the configuration for new treatment sites simple, as the only tuning required for plan recreation in Eclipse is of the priorities and normal tissue objective.

RapidPlan is a tool for automated planning already integrated in Eclipse, using so-called knowledge-based planning. Comparing the quality of iCE and RapidPlan plans was not within the scope of this study, in part because RapidPlan does not feature BAO. Studies have shown the dependence of the output plan quality on the quality of plans in the RapidPlan library [36,37]. In future work, we plan to explore the possibility of building a RapidPlan library containing iCE plans.

A limitation of the current study is the total number of investigated patients. However, all patients had LA-NSCLC and they were prospectively included. Moreover, there was a large variation in the tumor location and OAR dose-volume parameters among these LA-NSCLC patients. In order to strengthen the statistics, some patients were used for both Erasmus-iCycle wish-list tuning and for comparisons between CLIN and iCE. This is not a concern in the same way as for library-based methods, as the wish-list is based on the clinical protocol and priorities and not directly on the test patients used for guiding the wish-list creation.

Furthermore, some information from the clinical planning (choice of FB vs. DIBH technique and 60 Gy vs. 66 Gy prescription) was transferred to the automated planning, as required for fair comparison. These properties are sometimes changed during the planning process in response to the obtained OAR doses. With automated planning, several plans can be created with little additional work to find the most suitable option for each individual patient.

This study has been evaluated using the RATING criteria for treatment planning studies and a score of 96% was achieved [38].

## 5. Conclusions

The novel iCE system for automated multi-criterial IMRT treatment planning with integrated BAO was developed, and applied to optimize OAR sparing and to systematically investigate the impact of beam angles on plan quality for LA-NSCLC patients. A potential for improved sparing of the heart and esophagus was observed for most patients, with significant reductions in heart D_mean_ and V_30Gy_ and esophagus D_mean_ and V_20Gy_ with iCE compared to CLIN. Due to the automation, iCE reduced the hands-on planning time to the level of minutes. The dosimetric implications of increasing or reducing the number of treatment beams were highly patient-specific, but overall, increasing the number of beams improved OAR sparing. Due to the low workload, iCE could be used to generate a set of plans with different beam numbers for each patient and then select the best plan for the individual patient. Automated multi-criterial treatment planning with integrated BAO has great potential to further individualize and enhance radiotherapy for LA-NSCLC patients.

## Figures and Tables

**Table 1:** Planning dose constraints for the PTV, OARs and normal tissue. D_p_ = prescribed dose. In cases where fulfilling all constraints was impossible, the responsible oncologist decided whether target coverage or OAR constraints should be compromised.

Volume	Dose Constraint
PTV	V_95%_ > 98%
Lungs	V_5Gy_ < 65%
	V_20Gy_ < 35%
	D_mean_ < 20 Gy
Heart	V_30Gy_ < 40%
Esophagus	D_mean_ < 34 Gy
Spinal canal	D_max_ < 50 Gy
Brachial plexus	D_max_ < 66 Gy
Patient body	D_max_ < D_p_ · 1.07

**Table 2:** Applied Erasmus-iCycle wish-list. D_p_ = prescribed dose, 60/66/70 Gy; different logarithmic tumor control probability (LTCP) settings were used depending on D_p_. Note that the goal value for constraints was set slightly stricter than the actual clinical limit to account for finite sampling resolution.

Priority	Volume	Type	Goal	Sufficient
Constraint	PTV	Max	D_p_ · 1.02	
Constraint	PTV	Mean	D_p_ · 0.997	
Constraint	Spinal canal	Max	47 Gy	
Constraint	Brachial plexus	Max	60 Gy	
Constraint	Shell PTV + 1 cm ^1^	Max	D_p_	
Constraint	Shell PTV + 7 cm ^1^	Max	D_p_ · 0.75	
Constraint	External ring ^2^	Max	D_p_ · 0.8	
1	PTV	↓ LTCP ^3^	0.14/0.12/0.12	0.14/0.12/0.12
2	Lungs	↓ Mean	19 Gy	
3	Shell PTV + 3 mm ^1^	↓ Max	D_p_	D_p_
4	Shell PTV + 1 cm ^1^	↓ Max	D_p_ · 0.9	D_p_ · 0.9
5	Shell PTV + 7 cm ^1^	↓ Max	D_p_ · 0.65	
6	Lungs	↓ Mean	13 Gy	
7	Heart	↓ Mean	13 Gy	
8	Esophagus	↓ Mean	18 Gy	
9	Shell PTV + 3 cm ^1^	↓ Max	D_p_ · 0.75	
10	Shell PTV + 7 cm ^1^	↓ Max	D_p_ · 0.55	
11	Lungs	↓ Mean	0 Gy	
12	Heart	↓ Mean	0 Gy	
13	Esophagus	↓ Mean	0 Gy	

**^1^** Shells consist of all pixels located at the specified distance from the PTV. **^2^** Ring structure extending 2 cm inside the patient surface, subtracting PTV + 4 cm. **^3^** Logarithmic tumor control probability (LTCP) as defined in [10], using prescription D_p_ · 0.95 and α = 0.85/0.8/0.8.

**Table 3:** Applied Eclipse objective template. p.s. = patient specific, defined based on Erasmus-iCycle DVHs.

Volume	Type	Dose [Gy]	Priority
PTV_60/66/70	Min	59.5/65.5/69.5	130
	Max	60.5/66.5/70.5	130
Lungs	Line	p.s.	80
Heart	Line	p.s.	80
Esophagus	Line	p.s.	60
Spinal canal	Max	48	100
	Line	p.s.	40
Brachial plexus	Max	62	100
	Line	p.s.	40
Patient body	NTO ^1^	-	100

**^1^** NTO = normal tissue objective, with the following fixed parameters: distance from target border 0.5 cm, start dose 105%, end dose 60% and fall-off 0.15 [30].

**Table 4:** Patient and treatment characteristics.

Characteristic	Number of Patients
**Stage**	
IIB	2
IIIA	11
IIIB	10
IIIC	2
IVA	1
**Primary tumor location (lobe)**	
Right upper	10
Right upper + middle	1
Right lower	4
Left upper	4
Left lower	6
**Prescribed dose**	
60 Gy	10
66 Gy	15
70 Gy	1
**Chemotherapy**	
Concurrent	25
Sequential	1

**Table 5:** Dose-volume metrics for CLIN compared with iCE and iCE_noBAO plans. Median value and interquartile range (IQR) is given, along with *p*-values for difference with regard to CLIN. Significant differences from CLIN are marked with *.

Dose Metric	CLIN		iCE			iCE_noBAO		
	Median	IQR	Median	IQR	*p*	Median	IQR	*p*
PTV V_95%_ [%]	99.0	0.9	99.2	0.5	0.1	99.3	0.5	0.2
Lungs D_mean_ [Gy]	14.7	4.2	14.2	4.7	0.3	14.6	4.4	0.4
Lungs V_5Gy_ [%]	55.8	11.7	54.7	10.9	0.6	57.4	12.7	0.1
Lungs V_20Gy_ [%]	25.0	6.7	24.4	7.9	0.5	24.3	7.0	0.2
Heart D_mean_ [Gy]	9.0	7.1	8.1	5.9	0.02 *	8.3	6.7	0.07
Heart V_5Gy_ [%]	34.0	33.1	31.8	30.9	0.7	33.2	31.6	0.9
Heart V_30Gy_ [%]	11.0	9.5	6.2	6.5	0.002 *	8.8	8.4	0.002 *
Esophagus D_mean_ [Gy]	20.3	8.2	18.5	9.2	0.02 *	18.9	9.1	0.05 *
Esophagus V_20Gy_ [%]	38.4	14.3	36.8	17.7	0.008 *	35.4	16.4	0.08
Esophagus V_60Gy_ [%]	5.1	14.3	4.9	12.8	1.0	4.7	13.2	0.05 * ^1^

**^1^** Although the median value is lower, this parameter was significantly *increased* with iCE_noBAO compared to CLIN (the value was higher in iCE_noBAO for 16 patients, higher in CLIN for 9 patients and equal for 1 patient).

**Table 6:** Median differences in D_mean_ with range for OARs in 4- and 8-beam iCE plans compared to 6-beam iCE plans, all with optimized beam angles. *p*-values for comparison with 6-beam plans are given, and significant differences are marked with *.

Dose Metric	Difference, 4 vs. 6 Beams			Difference, 8 vs. 6 Beams		
	Median	Range	*p*	Median	Range	*p*
Lungs D_mean_ [Gy]	0.2	−2.5, 2.3	0.5	−0.2	−0.8, 0.5	0.004 *
Heart D_mean_ [Gy]	0.7	−2.3, 4.0	0.007 *	−0.3	−3.9, 1.5	0.02 *
Esophagus D_mean_ [Gy]	1.2	−4.2, 6.8	0.02 *	−0.7	−2.8, 2.9	<0.001 *

## Data Availability

The data presented in this study are available on request from the corresponding author. The data are not publicly available due to privacy reasons as they are part of an ongoing study.

## Supplementary Materials

The following are available online at https://www.mdpi.com/article/10.3390/cancers13225683/s1: Eclipse objective template: emptyObjectiveTemplate.xml and script for transferring patient-specific values from DVHs to objective templates: addObjectives.py.

## Appendix B. Impact of Applied Number of Beams per Patient

**Table A1:** Difference per patient in D_mean_ for OARs in 4- and 8-beam iCE plans compared to 6-beam iCE plans, all with optimized beam angles. The colors indicate if the dose was increased (red), reduced (green) or equal (yellow) compared to the 6-beam plan. For patients marked with *, the D_max_ requirement for the spinal canal or patient body was not met in the 4-beam plan only.

Patient	D_mean_ Difference [Gy], 4 vs. 6 Beams			D_mean_ Difference [Gy], 8 vs. 6 Beams		
	Lungs	Heart	Esophagus	Lungs	Heart	Esophagus
1	−2.5	1.8	3.3	0.5	−1.1	−2.3
2 *	0.6	−0.8	1.2	−0.3	1.5	−1.5
3 *	−2.0	0.6	6.8	0.3	−0.1	−2.8
4	0.7	0.8	4.6	−0.2	−0.8	2.9
5	0.4	1.3	2.3	−0.3	−0.9	−0.4
6	1.3	2.6	2.4	−0.4	1.1	−0.9
7 *	−0.6	0.0	−0.9	−0.4	0.0	−0.4
8	1.2	1.7	−0.1	−0.4	−0.5	−1.2
9	−0.1	−0.8	4.4	−0.2	−1.5	−0.1
10	−0.5	1.8	−1.4	0.0	−1.1	−0.2
11 *	−1.1	0.6	4.8	0.4	−0.4	−2.5
12	0.3	0.6	0.7	−0.4	−0.3	−1.4
13	0.9	0.9	3.4	−0.3	0.4	−1.4
14	2.0	3.2	−0.9	−0.6	0.0	−1.1
15 *	1.2	1.6	−2.2	−0.2	−0.1	−0.6
16	0.8	0.1	1.6	−0.2	−1.0	−1.8
17	−0.5	1.1	1.1	0.0	0.1	−0.3
18	−0.8	0.6	−0.8	−0.6	−0.5	−0.9
19	−0.3	0.3	0.1	0.0	−0.4	−0.2
20	−0.1	4.0	−4.2	−0.1	−0.2	−0.8
21 *	0.0	−2.3	4.5	−0.6	−3.9	0.3
22 *	0.8	−0.3	1.3	−0.8	−0.1	−0.6
23 *	−0.2	0.2	1.5	−0.2	−0.3	−0.5
24	1.8	3.5	−1.0	−0.2	−0.9	−0.9
25	2.3	1.9	0.0	0.2	−1.1	−0.3
26 *	−2.0	−1.8	−0.5	−0.4	0.7	−0.4
